# *Shigella flexneri* Infection in *Caenorhabditis elegans:* Cytopathological Examination and Identification of Host Responses

**DOI:** 10.1371/journal.pone.0106085

**Published:** 2014-09-04

**Authors:** Divya T. George, Carolyn A. Behm, David H. Hall, Ulrike Mathesius, Melanie Rug, Ken C. Q. Nguyen, Naresh K. Verma

**Affiliations:** 1 Division of Biomedical Science and Biochemistry, Research School of Biology, The Australian National University, Canberra, Australia; 2 Center for *C. elegans* Anatomy, Department of Neuroscience, Albert Einstein College of Medicine, Bronx, New York, United States of America; 3 Division of Plant Science, Research School of Biology, The Australian National University, Canberra, Australia; 4 Centre for Advanced Microscopy, The Australian National University, Canberra, Australia; New York State Dept. Health, United States of America

## Abstract

The Gram-negative bacterium *Shigella flexneri* is the causative agent of shigellosis, a diarrhoeal disease also known as bacillary dysentery. *S. flexneri* infects the colonic and rectal epithelia of its primate host and induces a cascade of inflammatory responses that culminates in the destruction of the host intestinal lining. Molecular characterization of host-pathogen interactions in this infection has been challenging due to the host specificity of *S. flexneri* strains, as it strictly infects humans and non-human primates. Recent studies have shown that *S. flexneri* infects the soil dwelling nematode *Caenorhabditis elegans,* however, the interactions between *S. flexneri* and *C. elegans* at the cellular level and the cause of nematode death are unknown. Here we attempt to gain insight into the complex host-pathogen interactions between *S. flexneri* and *C. elegans.* Using transmission electron microscopy, we show that live *S. flexneri* cells accumulate in the nematode intestinal lumen, produce outer membrane vesicles and invade nematode intestinal cells. Using two-dimensional differential in-gel electrophoresis we identified host proteins that are differentially expressed in response to *S. flexneri* infection. Four of the identified genes, *aco-1, cct-2, daf-19* and *hsp-60*, were knocked down using RNAi and ACO-1, CCT-2 and DAF-19, which were identified as up-regulated in response to *S. flexneri* infection, were found to be involved in the infection process. *aco-1* RNAi worms were more resistant to *S. flexneri* infection, suggesting *S. flexneri*-mediated disruption of host iron homeostasis. *cct-2* and *daf-19* RNAi worms were more susceptible to infection, suggesting that these genes are induced as a protective mechanism by *C. elegans*. These observations further our understanding of the processes involved in *S. flexneri* infection of *C. elegans,* which is immensely beneficial to the routine use of this new *in vivo* model to study *S. flexneri* pathogenesis.

## Introduction

Shigellosis, more commonly known as bacillary dysentery, is caused by enteric bacteria belonging to the genus *Shigella*. *Shigella flexneri* strains most frequently associated with the disease invade the colonic and rectal epithelia of their host and induce a strong inflammatory response that culminates in severe tissue damage; this manifests in a spectrum of clinical symptoms ranging from watery diarrhoea to severe dysentery characterized by fever, abdominal cramping and bloody, mucoid stool [1]. *S. flexneri* is highly contagious, with as few as 10–100 bacterial cells capable of causing infection [2]. The low infection dose, coupled with the emergence of numerous multidrug resistant strains of *S. flexneri,* has escalated the need to develop effective preventive and therapeutic measures to reduce the global burden of shigellosis.

*S. flexneri* has a very narrow host range and only infects human and non-human primate hosts, as a result of which there is no simple intestinal small-animal model available. The lack of a relevant *in vivo* model of shigellosis has been one of the major impediments to the development of preventive and therapeutic measures. A number of alternative animal models have been identified which use mucosal surfaces other than the colon as sites of infection. The most commonly used *in vivo* models are the murine pulmonary model of shigellosis [3]–[5] and the guinea pig keratoconjunctivitis model [6], however both these *in vivo* models lack clinical relevance as the site of *S. flexneri* infection and symptoms produced do not mirror infection in humans.

In recent years, the soil-dwelling roundworm, *Caenorhabditis elegans,* has been used extensively to study host-pathogen interactions *in vivo,* uncovering a wealth of information about microbial virulence factors and host defense responses [7]. *C. elegans* has been identified as a valuable *in vivo* model to study host-pathogen interactions on account of innumerable experimental advantages [8], [9]. This *in vivo* model is particularly useful to study enteric pathogens, as nematode intestinal cells share morphological similarities with human intestinal cells, including apical, finger-like microvilli anchored into a cytoskeletal terminal web composed of actin and intermediate filaments. In addition, the human innate immune system shares many characteristics with that of *C. elegans* and thus mechanisms of bacterial and nematode responses may be similar in mammalian cells [10]. A range of bacterial virulence factors have been shown to be required for both nematode and mammalian pathogenesis [8], [9], [11]–[14], further validating the use of *C. elegans* as a relevant *in vivo* model to study host-pathogen interactions. On account of these characteristics, the list of bacterial pathogens that are known to infect *C. elegans* is growing and includes prominent human pathogens such as *Salmonella enterica, Pseudomonas aeruginosa* and *Serratia marcescens* [15]–[17].

In the past decade, two independent groups have provided preliminary evidence to suggest that *C. elegans* can potentially be used as an *in vivo* model for shigellosis [18], [19]. These studies demonstrate that *S. flexneri* kills *C. elegans* in an infection-like process that requires live bacterial cells harboring intact virulence plasmids. Both studies also show that *S. flexneri* accumulates in the *C. elegans* intestine and kills the nematodes on solid media and in liquid culture. However, *C. elegans* as a model for shigellosis has not been completely understood, as the *S. flexneri-*mediated killing response and the nematode responses to *S. flexneri* infection remain unknown. This study aims to further our understanding of the interactions between *S. flexneri* and *C. elegans* in order to establish this *in vivo* model as a viable alternative to study *S. flexneri* pathogenesis. Here we report for the first time, the cytopathological changes induced in the nematode intestines during *S. flexneri* infection and identify novel host genes that are induced in response to *S. flexneri* infection.

## Results

### Wild type *S. flexneri* serotype 3b kills *C. elegans* and killing requires the expression of bacterial virulence plasmid-encoded genes

**Figure 1:**
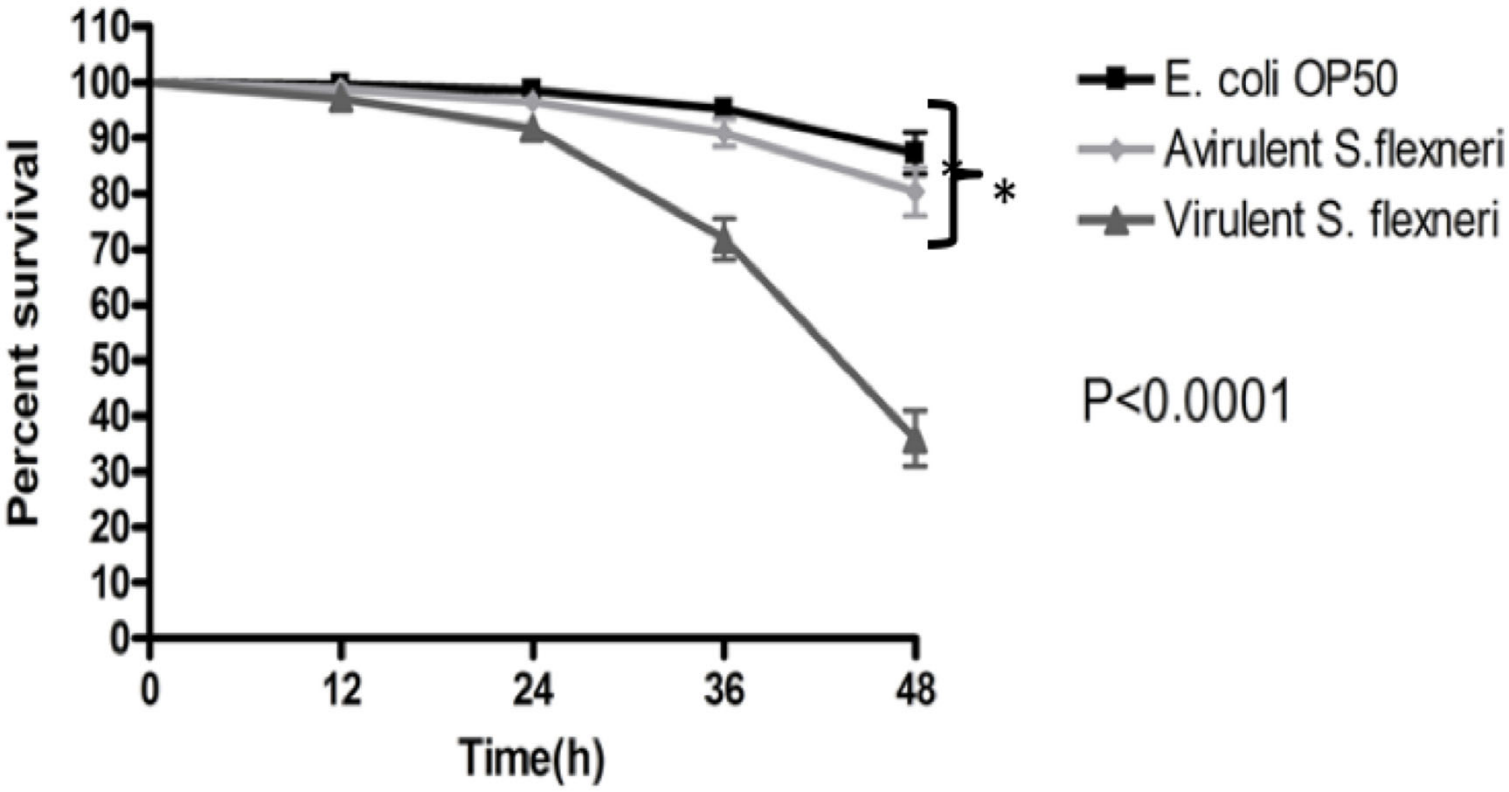
*S. flexneri* is pathogenic to *C. elegans*. Survival of wild-type N2 nematodes when fed *E. coli* OP50, an avirulent *S. flexneri* strain (virulence plasmid-cured *S. flexneri-*SFL1223), and virulent *S. flexneri* serotype 3b strain (SFL1520) (*p*<0.0001, Log rank test). Survival curve represents the means of data from three independent experiments with standard error (error bars), each using 20 nematodes. Asterisks indicate statistically significant differences.

Previously, Burton *et al.* [18] and Kesika *et al.* [19] have shown that wild type strains of *S. flexneri* serotypes 2a and 2b kill *C. elegans.* In this study, we chose to use the *S. flexneri* serotype 3b strain (SFL1520), identified to be virulent in the murine pulmonary model of shigellosis, to characterize the pathogenesis of this strain in *C. elegans.* Using liquid infection assays [19], we compared the survival rates of nematodes fed *S. flexneri* serotype 3b (SFL1520), an avirulent *S. flexneri* strain (which does not carry the virulence plasmid harboring genes required for adherence and invasion of host tissues) (SFL1223) and *E. coli* OP50 for 48 hours. Liquid killing assays were not carried out beyond 48 hours with N2 *C. elegans* due to the large number of L1/L2 progeny produced after 48 hours which masked the adults, thereby making scoring of survival rates difficult. Future studies using temperature-sensitive fertility mutants of *C. elegans* as hosts will allow investigation of the course of the infection beyond 48 hours. Results of the liquid killing assays with N2 worms showed that *S. flexneri* serotype 3b kills nematodes (TD_50_ = 46±1 h, where TD_50_ is the time taken to kill 50% of the initial worm population) much faster than control worms fed *E. coli* OP50 (<50% killing in 48 h) (Figure 1). Consistent with previous studies, we found that *S. flexneri* requires the virulence plasmid-encoded factors for *C. elegans* killing, as the survival of worms fed SFL1520 (carrying an intact virulence plasmid) was significantly reduced compared with worms fed SFL1223 (Figure 1).

### Wild type *S. flexneri* accumulates in the nematode intestinal lumen

**Figure 2:**
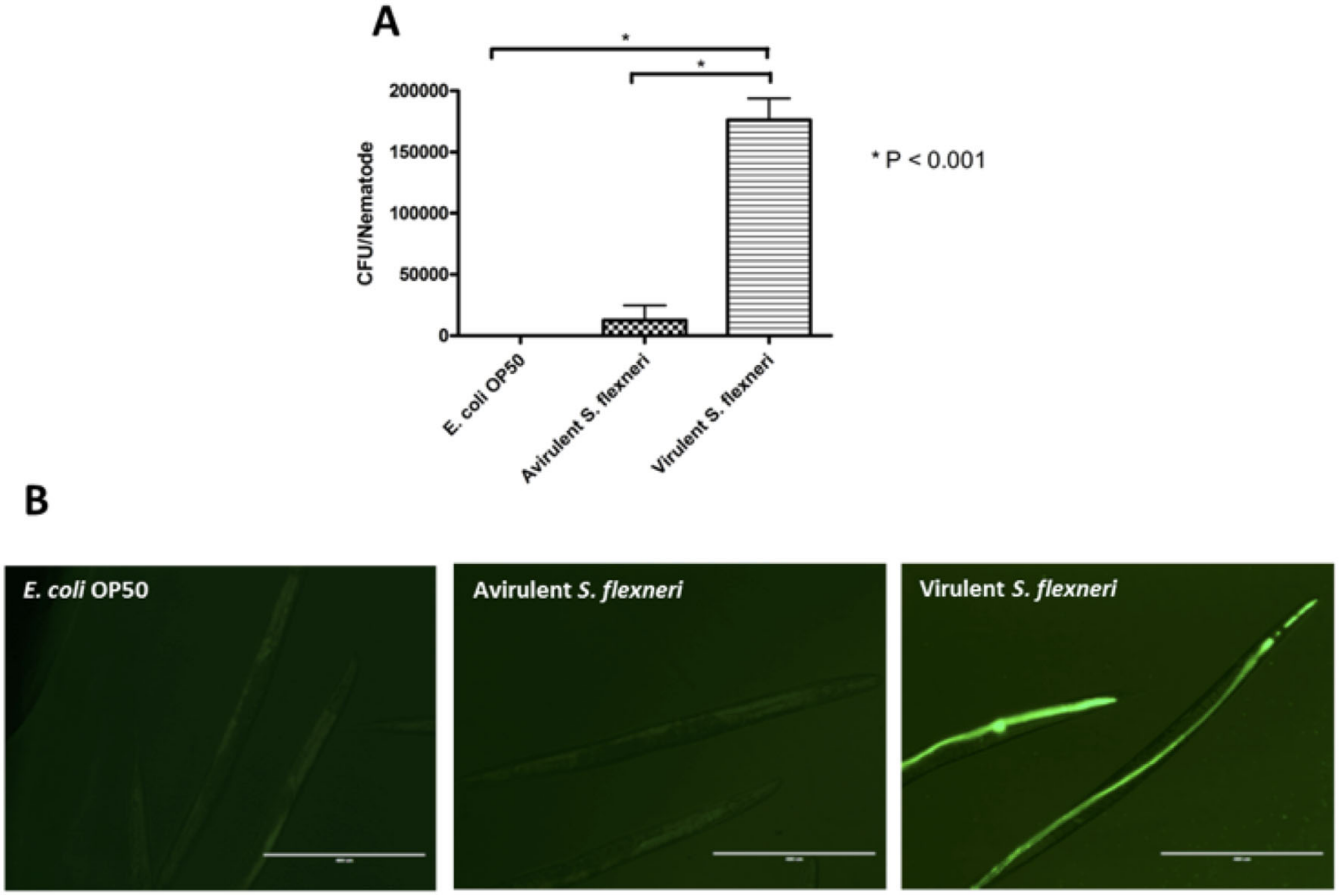
Virulent *S. flexneri* serotype 3b accumulates in *C. elegans* intestinal lumen 24 hours post infection. **A:** Young adult hermaphrodite N2 nematodes were fed either *E. coli* OP50, avirulent *S. flexneri* (SFL1223) or virulent *S. flexneri* 3b (SFL1520) for 24 hours at 22°C. 20 worms were picked off each bacterial lawn, disrupted using glass beads and appropriate dilutions of each lysate were plated on LB agar to obtain bacterial counts. Wild type *S. flexneri* 3b (SFL1520) accumulated in the intestinal lumen while the plasmid-cured *S. flexneri* strain (SFL1223) and OP50 were digested (*p*<0.001, One way ANOVA). Results represent the means of three independent experimental repeats with standard errors (error bars). **B:** Young adult hermaphrodite N2 nematodes were fed on lawns of *E. coli* OP50, avirulent *S. flexneri* (SFL1223) or virulent *S. flexneri* 3b (SFL1520) tagged with GFP^+^ for 24 h and fluorescence was observed using an EVOS digital inverted microscope (AMG). Scale bar = 400 µm.

Burton and colleagues [18] have previously shown that virulent *S. flexneri* serotype 2a cells accumulate in the intestinal lumen of nematodes, while avirulent *S. flexneri* strains are digested. To confirm that the *S. flexneri* serotype 3b strain also accumulates in the nematode intestinal lumen, we performed bacterial accumulation assays using wild type *S. flexneri* 3b (SFL1520), avirulent *S. flexneri* (SFL 1223), and *E. coli* OP50 as a negative control. The results of bacterial accumulation assays confirmed that *S. flexneri* serotype 3b-mediated killing of nematodes is associated with bacterial accumulation in the intestinal lumen (Figure 2.A). Using GFP^+^-tagged bacterial strains (bacterial strains transformed with plasmid pCR2.1, expressing GFP^+^), we further confirmed that wild type *S. flexneri* serotype 3b (SFL1520) accumulates in the nematode intestinal lumen while the avirulent strain (SFL1223) and *E. coli* OP50 are digested (Figure 2.B).

**Figure 3:**
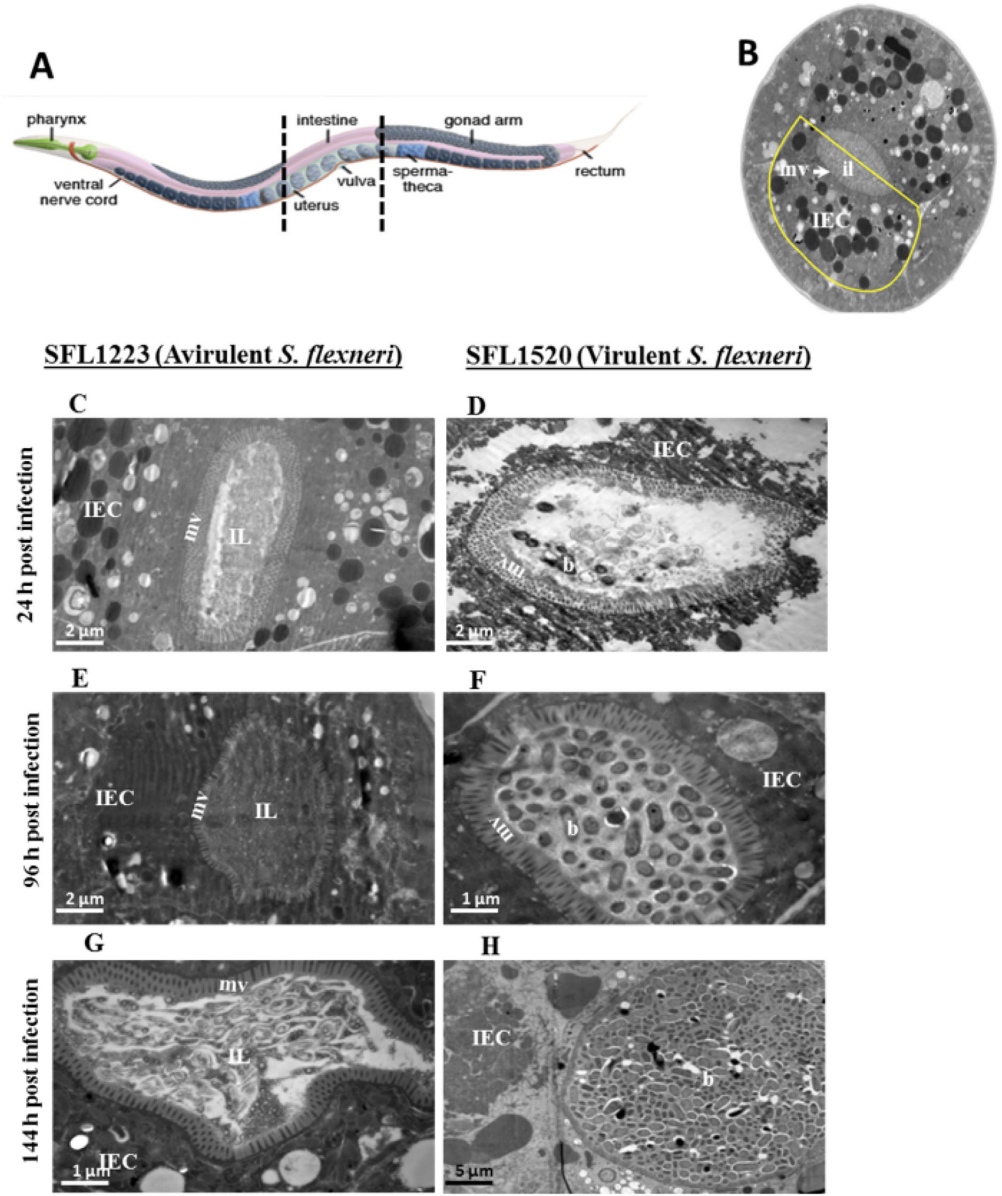
Virulent *S. flexneri* cells escape pharyngeal grinding and accumulate in the *C. elegans* intestinal lumen. **A:** Schematic representation of the *C. elegans* body plan with the plane of sections highlighted (artwork by Altun and Hall, © Wormatlas). **B:** Transverse section of the mid body of a healthy nematode with the intestinal cell highlighted in yellow. **C–H:** Transmission electron microscopy micrographs of transverse mid body sections of animals feeding on plasmid-cured, avirulent *S. flexneri* (SFL1223) (**C, E, G**) and virulent *S. flexneri* serotype 3b (SFL1520) (**D, F, H**) for 24 h (**C, D**), 96 h (**E, F**) and 144 h (**G, H**). IEC-intestinal epithelial cell; mv-microvilli; IL-intestinal lumen; b-intact *S. flexneri* cells.

A recent study showed that fluorescence produced by live and dead bacteria in the nematode intestinal lumen is indistinguishable [20]. Furthermore, the bacterial accumulation assays fail to distinguish between live bacterial accumulation in the pharynx and bacterial cells that have progressed past the grinder in the terminal bulb. Thus, the results of bacterial accumulation observed using the accumulation assay and fluorescence assays fail to confirm that live bacterial cells evade grinding by the nematode grinder and accumulate in the intestinal lumen. We therefore used transmission electron microscopy (TEM) to compare the intestinal lumen of nematodes fed avirulent *S. flexneri* SFL1223 and wild type *S. flexneri* serotype 3b (SFL1520) over three time points, 24, 96 and 144 hours post infection. Using this approach we confirmed the presence of intact *S. flexneri* serotype 3b (SFL1520) cells in the intestinal lumina of infected nematodes, with the bacterial load increasing over time (Figure 3D, F and H). No intact bacterial cells were observed in the intestinal lumina of nematodes fed the avirulent strain (Figure 3C, E and G). These observations clearly suggest that virulent *S. flexneri* cells escape the grinder-mediated breakdown and accumulate in the *C. elegans* intestinal lumen.

### Intraluminal *S. flexneri* cells produce putative outer membrane vesicles (OMVs) and invade *C. elegans* intestinal cells

**Figure 4:**
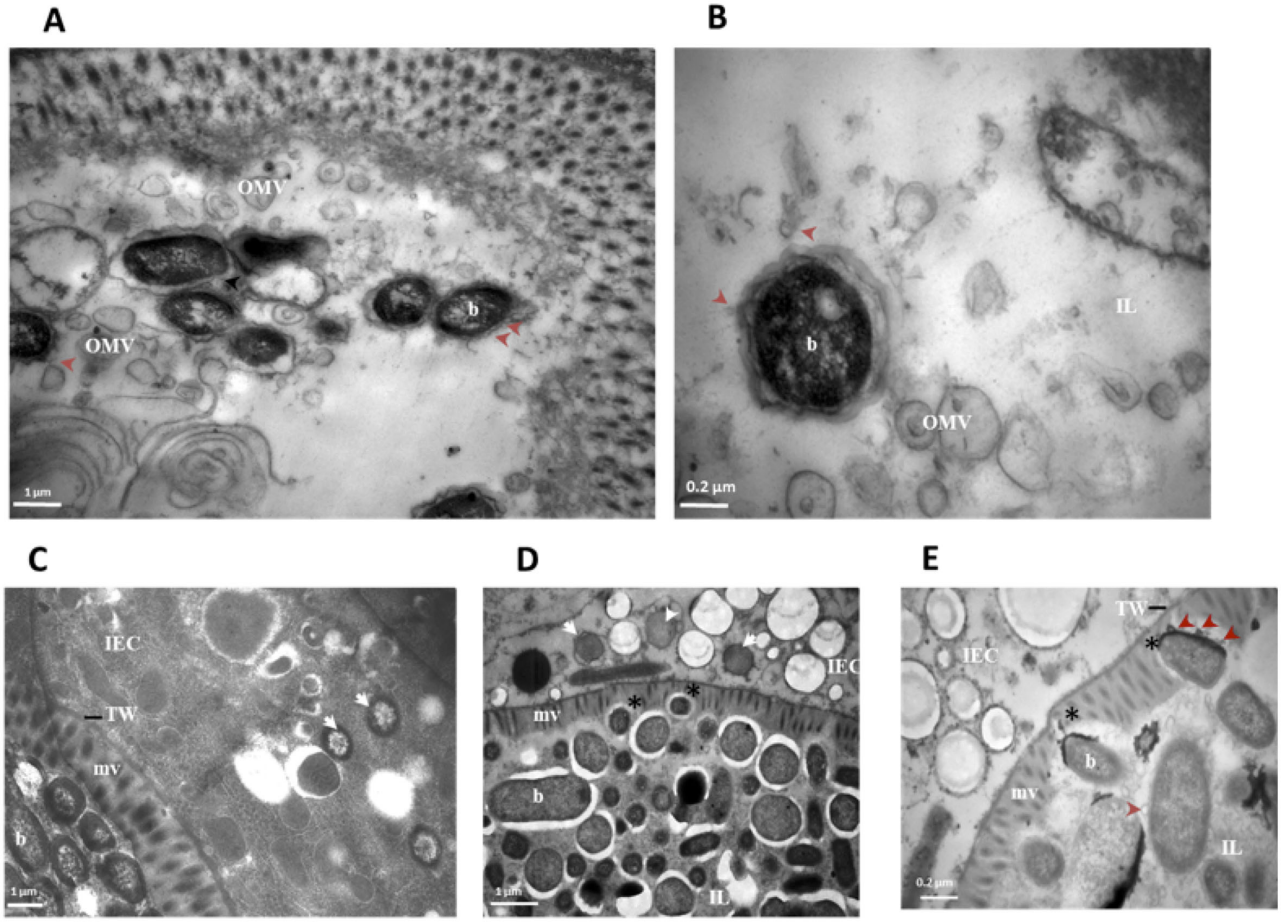
Intraluminal *S. flexneri* cells produce putative outer membrane vesicles and invade *C. elegans* intestinal cells. **A–E:** TEM micrographs of transverse mid body sections of young adult nematodes infected with wild type *S. flexneri* serotype 3b (SFL1520) for 24 h (**A, B**), 96 h (**C**) and 144 h (**D, E**). **A, B:** Intraluminal bacterial cells produce outer membrane vesicles (OMV), red arrowheads indicate OMV shedding from bacterial cells. **C, D:** White arrowheads indicate intracellular bacterial cells that have penetrated the ciliated epithelial barrier of the intestinal cell, 96 and 144 h post infection. **E:** Intraluminal *S. flexneri* cells degrading the apical microvilli boundary (asterisk) of the *C. elegans* intestinal cells. IEC- intestinal epithelial cells; mv-microvilli; IL-intestinal lumen; TW-terminal web; b-intraluminal bacterial cells; OMV-outer membrane vesicles.

TEM micrographs revealed that *S. flexneri* serotype 3b cells within the nematode intestinal lumen produce putative outer membrane vesicles (OMVs) (Figure 4.A and B). OMVs are secreted elements produced by Gram-negative bacteria as part of a bacterial stress response induced during infection of host tissues [21]. *S. flexneri* OMVs contain the invasion protein antigens - Ipa proteins (IpaB, C & D) that are required for bacterial adherence, invasion and survival within infected tissues. It has been shown that the production of OMVs by *S. flexneri* cells provides a mechanism for the delivery of virulence factors to host tissues [22]. The identification of *S. flexneri* OMVs in the *C. elegans* intestinal lumen therefore suggests a potential mechanism for the delivery of bacterial virulence factors to host cells.

Using TEM, intracellular *S. flexneri* cells were observed 96 and 144 hours post infection (Figure 4.C and D). These observations indicated that virulent *S. flexneri* cells cross the protective, apical microvilli boundary of nematode intestinal cells and enter the intestinal cell cytoplasm. We also found evidence suggesting that intraluminal *S. flexneri* cells degrade the microvilli (Figure 4.E). These findings are extremely significant as, although several studies have shown that intracellular mammalian pathogens kill *C. elegans* by persistently colonizing the intestinal lumen, most of these pathogens remain extracellular and fail to invade the nematode intestinal cells [23].

### Identification of nematode responses to *S. flexneri* infection using two dimensional differential in-gel electrophoresis

Two dimensional differential in-gel electrophoresis (DIGE) was performed to gain insight into *S. flexneri-*induced responses in *C. elegans.* Total protein was isolated from nematodes infected with *S. flexneri* serotype 3b and *E. coli* OP50 for 24 hours. We decided to use a 24 hour infection period as we observed significant accumulation of *S. flexneri* in the worm intestine at this time point (Figure 2). 2 mg of total protein, isolated from four independent experiments, was used to perform DIGE. Analysis of DIGE results identified 41 *S. flexneri*-induced nematode proteins (37 up-regulated and 4 down-regulated) (Figure S1). All 41 identified spots were excised from the gels for identification by peptide sequencing using liquid chromatography mass spectrometry (LC-MS), following which, proteins were identified using the MASCOT database (Matrix Science).

MASCOT searches using stringent search parameters (mass tolerance between 0.3 and 0.1 Da), only identified 7 proteins out of 41 predicted protein spots (Figure S1 and Table 1). The low success rate of protein identification could be due to insufficient protein in the spots excised from the DIGE gels. Since MASCOT searches only identified 7 out of 41 potential *S. flexneri-*induced nematode proteins, the stringency of the search parameters was decreased in an attempt to identify the remaining proteins. Using low stringency MASCOT search parameters (mass tolerance between 0.8 Da and 0.6 Da), we identified significant hits for 39 out of the 41 identified spots, with many spots corresponding to more than one protein (Table S1). Some of these proteins (UNC-22, NHR-77 and C10C6.6) were identified more than once in different locations on the gel and thus represent different isoforms, which could be the result of post-translational modifications or different splice variants of the same protein. Theoretical molecular weights (Mwt) and pI values of identified proteins were compared with the Mwt and pI of spots on the DIGE gels; peptide coverage and distribution were also taken into account to eliminate false positive hits.

**Table 1:** Predicted *S. flexneri-*induced *C. elegans* responses using DIGE analysis.

Identifiedprotein	Genename	*Shigella*-inducedchange inexpression	Description	PredictedpI/Mwt (kDa)	ObservedpI/Mwt (kDa)	PeptideCoverage(%)	No ofpeptidesMatched	Score	P value(One WayANOVA)
Putative stonedB-like protein	*unc-41*	Down-regulated	Potential adapter protein,which may be involved inendocytic vesicle recyclingof synaptic vesicles	4.81/190.58	4.8/∼94.00	3	7	27	0.029
Myosin-4	*unc-54*	Up-regulated	Encodes a muscle myosinclass II heavy chain (MHC B).Expressed in the intestine.Involved in pharyngealpumping and egg laying	5.59/225.958	5.2/96.00	8	5	134	0.038
Probable cytoplasmicaconitate hydratase	*aco-1*	Up-regulated	Enzyme that catalyzes theisomerization of citrate toisocitrate via cis-aconitate.Required for iron homeostasis	5.49/97.11	5.5/95.00	14	5	86	0.022
Elongation factor 2	*eef-2*	Up-regulated	Catalyzes the GTP-dependentribosomal translocation stepduring translation elongation.Required for embryogenesis	6.1/95.47	5.5/95.00	18	8	72	0.022
RFX-liketranscription factor	*daf-19*	Up-regulated	A transcription factor thatregulates genes of ciliatedsensory neurons. *daf-19*mutants are defective in theirability to taste or smell and aresusceptible to bacterial infection!	5.97/91.42	5.1/86.00	2	3	24	0.040
Chaperonin homologHsp-60	*hsp-*60	Up-regulated	Heat shock protein implicated inmitochondrial proteinimport and macromolecularassembly. Required forresponse to oxidative stress	5.31/60.24	5.1/68.00	49	20	1446	0.040
T-complex protein1 subunit beta	*cct-2*	Up-regulated	Molecular chaperone; assiststhe folding of proteinsupon ATP hydrolysis.	5.65/53.38	5.6/67.5	6	2	41	0.047

Proteins identified as potential *S. flexneri-*induced nematode responses were subdivided into different categories based on their biological functions (Figure S2). Most of the nematode proteins induced by *S. flexneri* infection were involved in transcription and translation, followed closely by proteins required for locomotion and pharyngeal pumping.

### Quantitative real-time reverse-transcriptase PCR (qRT-PCR) to confirm the results of DIGE analysis

**Figure 5:**
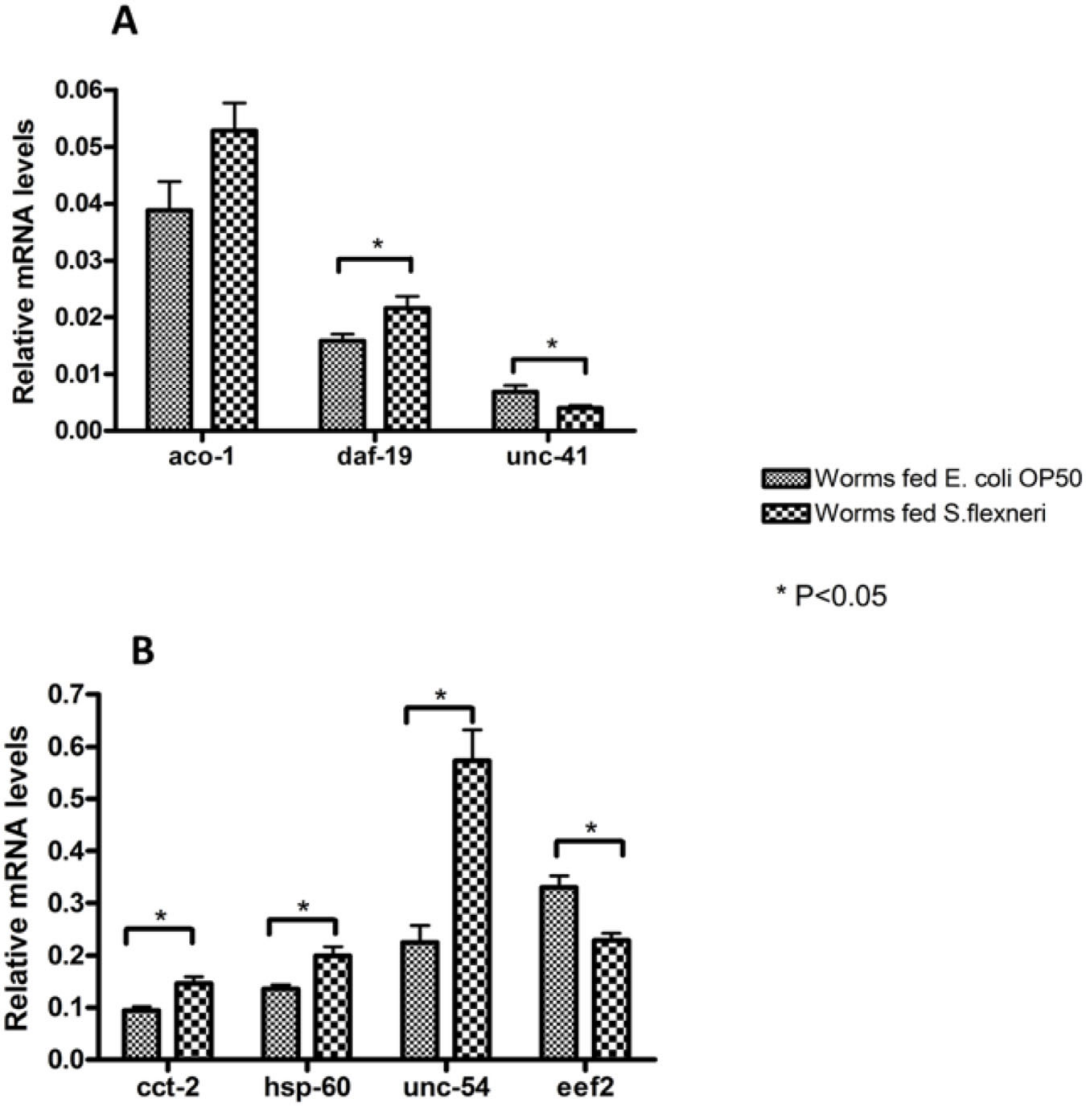
Reverse transcription quantitative PCR (qRT-PCR) analysis of *S. flexneri-*induced genes predicted by DIGE analysis. Transcript levels of *aco-1, daf-19* and *unc-41* (**A**), *cct-2, hsp-60, unc-54* and *eef-2* (**B**) were measured in synchronized young adult wild type animals feeding on *E. coli* OP50 or infected with *S. flexneri* serotype 3b for 24 h. Data represent the means of three biological replicates, each replicate measured in triplicate and normalized to the control gene, *act-*2, expressed as the ratio of the corresponding *S. flexneri-*induced levels and the basal *E. coli* OP50 levels. Asterisks indicate statistically significant differences identified using unpaired Student’s *t*-tests and error bars represent standard error.

Quantitative real-time reverse transcription polymerase chain reaction (qRT-PCR) was used to compare mRNA levels of the seven proteins identified using high stringency MASCOT search parameters (ACO-1, CCT-2, EEF-2, DAF-19, HSP-60, UNC-54 and UNC-41). The *C. elegans act-*2 gene, which encodes actin, was used as a control gene to normalize all reactions, as the mRNA levels of *act-*2 were expected to remain constant in both healthy and infected worms. Consistent with our DIGE analysis, qRT-PCR revealed a significant increase in the transcript levels of *cct-2, daf-19, hsp-60* and *unc-54* and a decrease in the level of *unc-41* in worms infected with *S. flexneri* serotype 3b (Figure 5). No statistically significant differences were observed in the transcript levels of *aco-1* in infected and control worms. *aco-1* encodes aconitase, an enzyme whose expression is regulated post-translationally by iron levels [24]. This would explain why the elevated levels of ACO-1 protein observed in response to *S. flexneri* infection were not reflected by the mRNA levels in infected versus uninfected worms. Contrary to the DIGE analysis, the transcription of *eef-2* appeared to be down-regulated, suggesting that the EEF-2 protein levels in infected worms may also be regulated post-transcriptionally. The changes in protein and transcript expression observed in the DIGE and qRT-PCR experiments described above should ideally be confirmed by Western analysis when suitable antibody preparations become available.

### RNAi-mediated knock down of *aco-1, cct-2, daf-19* and *hsp-60*

**Figure 6:**
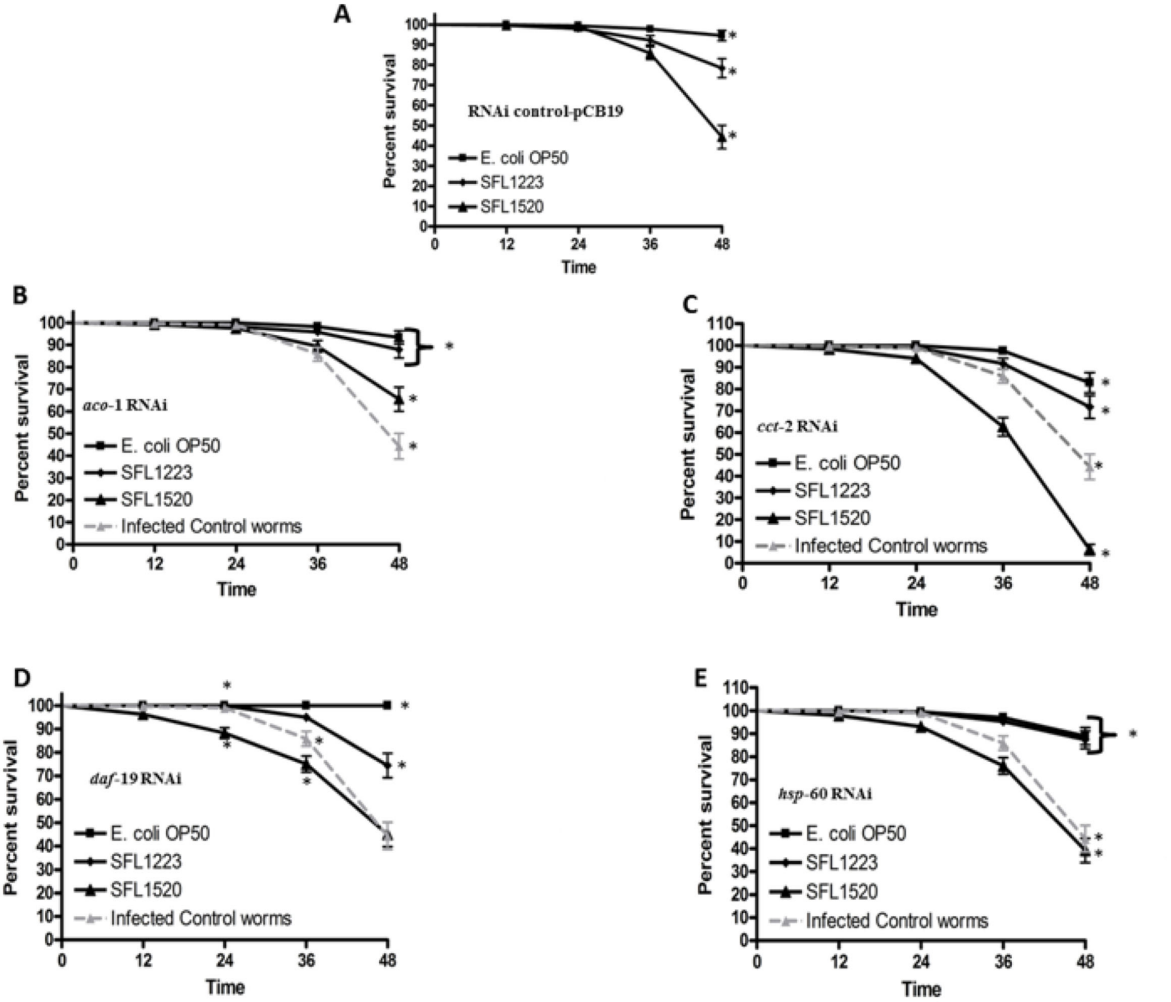
Effects of knockdown of *S. flexneri-*induced host response genes on nematode survival. After RNAi knockdown of *S. flexneri-*induced genes *aco*-1 (**B**), *cct*-2 (**C**), *daf-*19 (**D**) and *hsp-*60 (**E**), young adult hermaphrodites were transferred onto lawns of either *E. coli* OP50, avirulent *S. flexneri* (SFL1223) or virulent *S. flexneri* serotype 3b (SFL1520) and scored at 12 hour intervals for survival. **A:** Nematodes treated with pCB19 (RNAi control vector) were used as a non-specific ds-RNA control. Survival curve represents data from three independent experiments, each using 20 nematodes. The survival curves of RNAi worms infected with *S. flexneri* serotype 3b were compared with RNAi control-pCB19 worms infected with *S. flexneri* serotype 3b (grey, dashed curves); and asterisks indicate statistically significant difference in survival curves (*p*<0.05) identified using Log rank tests.

**Figure 7:**
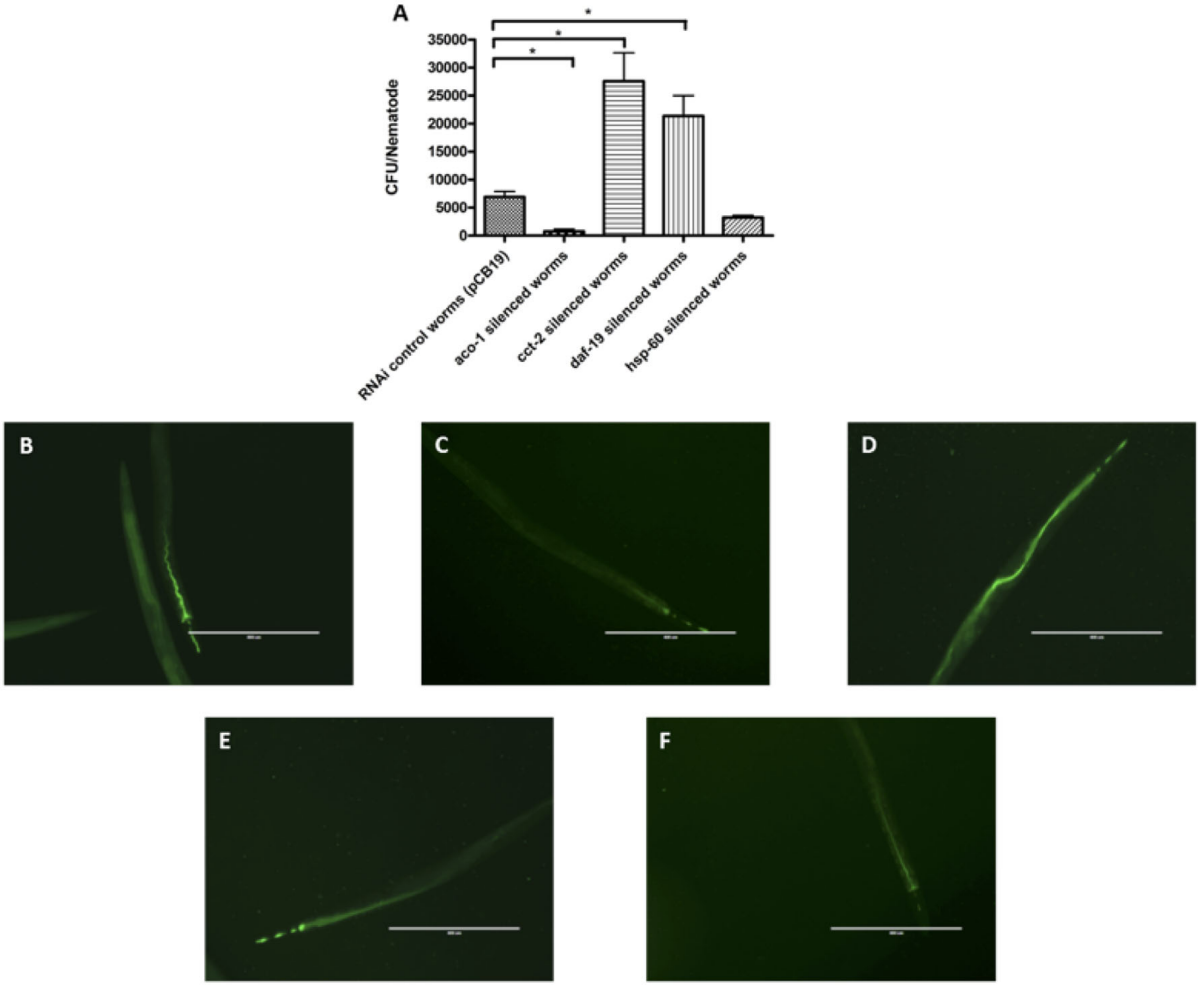
Effects of knockdown of *S. flexneri-*induced host response genes on bacterial accumulation. After RNAi knockdown of *S. flexneri*-induced genes, *aco-1, daf-19, hsp-60,* plus pCB19 as an RNAi negative control, young adult hermaphrodites were transferred onto lawns of virulent *S. flexneri* (SFL1520) for 24 h. **A:** 20 worms were picked off bacterial lawns and disrupted following which appropriate dilutions of each lysate was plated onto LB agar to obtain the bacterial counts. Results represent the means of three independent experimental repeats with standard error (error bars), asterisks indicate statistically significant differences (*p<*0.005, unpaired *t-*tests). pCB19 (**B**), *aco-1* (**C**), *cct-2* (**D**), *daf-19* (**E**) and *hsp-60* (**F**) RNAi knockdown animals were transferred onto GFP^+^-tagged SFL1520 for 24 h. Fluorescence was observed using the EVOS digital inverted microscope (AMG) and representative images are shown. Scale bar = 400 µm.

RNAi nematodes for *aco-1, cct-2, daf-19, hsp-60* and pCB19 (RNAi control) were prepared using the feeding protocol as described in materials and methods. Target genes were silenced in nematodes from the L1 stage using the appropriate RNAi constructs. Nematodes were maintained on *E. coli* lawns harboring the appropriate RNAi constructs until they reached the L4 stage, following which the RNAi worms were infected with *S. flexneri* serotype 3b (SFL1520), avirulent *S. flexneri* (SFL1223) and *E. coli* OP50 for 24–48 hours. Liquid killing (Figure 6.A-E) and bacterial accumulation (Figure 7.A–F) assays were performed to determine whether these *S. flexneri*-induced genes play a role in protecting nematodes against *S. flexneri* infection.

*aco-1* RNAi nematodes fed *S. flexneri* serotype 3b showed a significant increase in survival rates (with less that 50% killing in 48 h) compared with the RNAi control worms (TD_50_ = 46±1 h) (Figure 6.B). This enhanced resistance to *S. flexneri* serotype 3b infection correlated with decreased bacterial accumulation (Figure 7.A and C). These observations suggest that *aco-1* acts as a negative regulator of host responses to *S. flexneri* serotype 3b infection.

*cct*-2 RNAi nematodes fed *S. flexneri* serotype 3b were more susceptible to *S. flexneri* infection (TD_50_ 38±2 h compared with TD_50_ = 46±1 h of control worms, *p*<0.0001, Log rank test) (Figure 6.C). The increased killing of *cct*-2 RNAi worms correlates with increased bacterial accumulation (Figure 7.A and D). These observations suggest that CCT-2 induces or contributes to host protective mechanisms.

*daf-19* RNAi worms showed increased bacterial accumulation (Figure 7.A and E) and significantly enhanced susceptibility to *S. flexneri-*mediated killing at earlier time points when compared with RNAi control worms (24 and 36 hours, *p<*0.0001, Log rank test) (Figure 6. D). These observations suggest that DAF-19 is involved in the initial nematode protective mechanisms. *hsp-60* RNAi worms were slightly more susceptible to *S. flexneri-*mediated killing (TD_50_ 45±1) compared with control worms (TD_50_ 46±1 h, *p*<0.0416), however no significant differences were observed in *S. flexneri* accumulation in *hsp*-60 RNAi and control worms (Figure 7.A and F).

## Discussion

### Virulent *S. flexneri* cells ingested by *C. elegans,* evade pharyngeal grinding, accumulate in the nematode intestinal lumen and invade the intestinal cells

Consistent with previous reports, here we confirmed that a virulent *S. flexneri* serotype 3b strain kills nematodes and nematode killing was associated with bacterial accumulation within the intestinal lumen. TEM micrographs obtained in this study show the presence of intact *S. flexneri* cells within the intestinal lumina of infected worms, with the bacterial load increasing over time (Figure 3). These results suggest that *S. flexneri* cells evade pharyngeal grinding. The *C. elegans* grinder crushes ingested bacterial cells and is therefore the worm’s primary defense against invading pathogens. The presence of intact *S. flexneri* cells within the nematode intestinal lumen suggests that *S. flexneri* cells overcome this nematode defense mechanism.

Worms infected with virulent *S. flexneri* are sluggish, show impaired grinding of ingested food, and fail to lay eggs as efficiently as control worms [19]. All these symptoms correlate with an overall loss of energy and defective neuromuscular functions. Results of our DIGE analysis identified several nematode proteins involved in locomotion (UNC-22, UNC-41, UNC-54, UNC-79 and UNC-89) and pharyngeal pumping (UNC-54, SLO-1 and HSP-6) as differentially expressed in response to *S. flexneri* infection. These observations suggest that *S. flexneri* infection potentially induces an overall systemic neuromuscular defect in infected nematodes, leading to defective grinder activity.

TEM micrographs obtained in this study show that live *S. flexneri* cells within the nematode intestinal lumen produce outer membrane vesicles (OMVs) (Figure 4.A and B). OMVs produced by pathogenic bacteria contain biologically active proteins, including virulence factors, proteases and immunomodulatory compounds required for infection [25]–[31]. OMV production therefore constitutes a mechanism used by pathogenic bacteria to deliver virulence factors to host cells [32]–[36]. *S. flexneri* OMVs contain the invasion protein antigens-Ipa proteins (IpaB, C and D) that are required for bacterial adherence, invasion and survival within infected tissues [22]. OMVs produced by *S. flexneri* have been shown to adhere to and invade mammalian cells [22]. The identification of *S. flexneri* OMV in the *C. elegans* intestinal lumen therefore suggests a potential mechanism for the delivery of *S. flexneri* virulence factors to the nematode intestinal cells. This will be tested further by investigating the effects of exposing *C. elegans* to isolated *S. flexneri* OMVs.

TEM analysis also indicates that *S. flexneri* cells cross the protective apical microvilli brush border lining and invade the nematode intestinal cells. This finding is highly significant: while several intracellular bacterial pathogens have been shown to infect *C. elegans,* they remain extracellular throughout nematode infection, in contrast to the infection in mammals [23]. During human infection, *S. flexneri* cells exploit the transcytotic properties of specialized M cells to gain access into the sub-epithelial space, and invade the colonic and rectal epithelial cells through their less-protected basolateral surfaces [37]. *C. elegans* lacks specialized M cells and the results of this study suggest that *S. flexneri* possibly invade the nematode intestinal cells through the apical side by degrading the protective microvilli (Figure 4.E). A similar bacterial attack on intestinal microvilli (but without cytoplasmic invasion) has previously been noted in very old nematodes [38], perhaps due to changes in OP50 when they are allowed to continue growing in this environment, or due to a loss of innate immunity or structural integrity in extreme aging.

The ability of *S. flexneri* to invade the *C. elegans* intestinal cells makes this animal model a promising alternative to study shigellosis. Outside of Macaque monkeys, none of the current animal models of shigellosis show bacterial invasion of intestinal cells following oral infection.

### *S. flexneri* may disrupt iron homeostasis in infected cells and potentially induces a hypoxic response, resulting in nematode death

ACO-1 was shown to be up-regulated in response to *S. flexneri* infection. RNAi-mediated silencing of *aco-1* indicated that on knocking down this gene, worms showed enhanced resistance to *S. flexneri* infection. These results suggest that the up-regulation of ACO-1 in response to *S. flexneri* infection contributes to nematode death. *aco-1* encodes aconitase, an enzyme whose expression is negatively regulated post-translationally by iron levels [39]. In *C. elegans, aco-1* is expressed in the cytosol of cells of the hypodermis and intestine [24]. Its mammalian homologue, iron regulatory protein 1 (IRP-1), expressed in the brain and intestinal cells, plays a central role in iron homeostasis. The up-regulation of ACO-1 in response to *S. flexneri* infection therefore suggests that *S. flexneri* potentially disrupts iron homeostasis in *C. elegans.*

A recent study showed that *Pseudomonas aeruginosa* infection disrupts iron homeostasis in *C. elegans,* causing a *hif-1*-mediated hypoxic response leading to nematode death [40]. Similarly, *S. flexneri* could potentially kill nematodes by disrupting iron homeostasis, leading to the up-regulation of ACO-1 and the induction of a hypoxic response. Results of our DIGE analysis also identified the nematode MTSS1 protein (Table S1, spot no. 32) as down-regulated in response to *S. flexneri* infection. MTSS1 is a mitochondrial single stranded DNA binding protein that is essential for mitochondrial DNA replication [41]. RNAi knock down of MTSS1 results in the transcriptional alterations leading to the induction of hypoxia responses [41]. The up-regulation of ACO-1 and down-regulation of MTSS1 in response to *S. flexneri* suggests that *S. flexneri* potentially disrupts nematode iron homeostasis which potentially induces a hypoxic response culminating in death.

### Nematode innate immune responses to *S. flexneri* infection

RNAi experiments clearly indicated that *cct-2* and *daf-19* knock down worms show enhanced susceptibility to *S. flexneri* infection (Figures 6.C, D and 7.D, E), suggesting that these responses form part of the nematode protective mechanism. The chaperonin CCT-2 has been predicted to interact with DAF-16, which forms part of the nematode DAF-2/DAF-16 insulin signaling pathway; RNAi knock down of *cct-2* inhibits the nuclear localization of DAF-16 in intestinal cells [42], suggesting that CCT-2 is required for the nuclear translocation of DAF-16. DAF-16 is a transcription factor that has been associated with the expression of several antimicrobial genes [43]. The increased susceptibility of *cct-2* RNAi worms to *S. flexneri* infection could therefore be due to the decreased expression of antimicrobial peptides on account of defective nuclear translocation of DAF-16. The up-regulation of CCT-2 in response to *S. flexneri* and the increased susceptibility of *cct-2* RNAi worms suggest that *S. flexneri* infection activates the DAF-2/DAF-16 insulin signaling pathway.

DAF-19 is a transcription factor that has recently been shown to play a role in the p38 mitogen activated protein kinase (MAPK) pathway in *C. elegans* [44]. DAF-19 mutant worms display enhanced susceptibility to killing by *P. aeruginosa* [44]. DAF-19 is an ortholog of the regulatory factor X (RFX), a transcription factor that is required for human adaptive immunity [45]. The up-regulation of DAF-19 in *S. flexneri* infected worms could therefore be part of the nematode innate immune response at the early stages of *S. flexneri* infection. Furthermore, TIR-1 (Table S1, spot no. 7) was also identified to be up-regulated in response to *S. flexneri* infection using low stringency MASCOT searches. TIR-1 is the toll interleukin 1 receptor domain adaptor protein in *C. elegans* that activates the p38 MAPK innate immune response. During human infection, the multiplication of *S. flexneri* cells within infected intestinal cells activates the MAPK-mediated signaling pathway, thus leading to the production of inflammatory chemokines and cytokines and antimicrobial peptides [46], [47]. The up-regulation of both DAF-19 and TIR-1 in infected worms and increased susceptibility of *daf*-19 RNAi worms suggests that the p38 MAPK pathway is potentially induced by *C. elegans* in response to *S. flexneri* infection.

## Conclusion

Shigellosis research has been hindered by the lack of an appropriate *in vivo* model. Unlike humans and non-human primates, the small animal models of shigellosis fail to develop intestinal disease upon ingestion of *S. flexneri.* Here we have shown that virulent strains of *S. flexneri* are ingested by the nematode *C. elegans* and that *S. flexneri* cells invade the nematode intestinal cells. This is the first report to investigate the cytopathology and nematode responses to *S. flexneri* infection. DIGE analysis of the nematode proteome induced by *S. flexneri* suggests that both the DAF-2/DAF-16 insulin signaling pathway and the p38 MAPK pathway are induced in response to bacterial infection. Our findings also suggest that *S. flexneri* disrupts iron homeostasis in nematodes and potentially induces a hypoxic response which could lead to death. The results of this study further our understanding of *S. flexneri* infection in *C. elegans,* opening up a new, convenient, economical alternative for screening *Shigella* mutant strains to identify attenuated strains for testing live vaccine strains and for the identification of novel virulence factors.

## Materials and Methods

### Nematode and bacterial strains

*C. elegans* strain Bristol N2 [48] obtained from the *Caenorhabditis* Genetic Center (CGC), Minneapolis, MN, was cultured and maintained at 22°C on modified nematode growth medium agar [18] and fed with *E. coli* OP50 as described previously [49]. RNAi of *aco-1, cct-2, daf-19,* and *hsp-60* was performed by feeding as described previously [50]. *E. coli* HT115 (DE3) harboring genes encoding double stranded RNA targeted towards *aco-1* (ZK455.7), *cct-2* (T21B10.7), *eff-2* (F25H5.4) and *hsp-60* (Y22D7AL.5) were obtained from the Ahringer lab library and the *daf-19* (F33H1.1) RNAi construct was obtained from the ORF-RNAi Library v1.1 (Source Bioscience). pCB19 was used as a non-specific dsRNA control, as this vector contains a fragment of the *Arabidopsis thaliana* light harvesting complex gene (LHCB4.3), which shows no homology to *C. elegans,* cloned into the RNAi vector pL4440 [51].

Liquid overnight cultures of bacteria were grown in Luria Bertani (LB) broth, containing ampicillin (100 µg/mL) when necessary. The virulent *S. flexneri* serotype 3b strain-SFL1520 (International Centre for Diarrhoeal Diseases Research Bangladesh-ICDDRB) and virulence plasmid-cured avirulent strain-SFL1223 [52] were used. *S. flexneri* cultures were grown at 30°C overnight for maintenance of the virulence plasmid, following which bacterial cultures were grown to log phase at 37°C to induce expression of the plasmid-based virulence genes.

### *C. elegans* liquid killing assays

Killing assays in liquid culture were performed as described previously [19]. Briefly, a synchronized population of young adult *C. elegans* worms was treated with 200 µg/mL gentamycin for 3 hours to eliminate any surface-bound bacteria. Worms were washed thoroughly with S-basal to remove any residual antibiotic. Approximately 20 washed young adults were transferred into each well in a 24-well plate containing 100 µL of appropriate log-phase *S. flexneri* (OD_600_ = 0.6) and *E. coli* cultures (OD_600_ = 0.5). The volume of solution in each well was adjusted to 500 µL with S-basal and plates were incubated at 22°C. The number of live worms in each well was scored every 12 hours and percentage survival was calculated. Nematodes that showed no pharyngeal pumping and remained immobile on tapping the plate were considered dead. Results are representative of three independent assays, each with triplicates. Survival curves were analysed using the PRISM (version 4.02) software. Kaplan-Meier analysis was used to compare the mean lifespan of *C. elegans.* Logrank tests were used to determine if survival curves were significantly different.

### Bacterial accumulation assay

Bacterial strains used for the accumulation assay were grown overnight at 37°C on modified NG agar medium to stimulate expression of virulence plasmid-encoded genes [18]. Plates were cooled to room temperature before they were inoculated with 50–100 synchronized young adult nematodes and incubated at 22°C for 24 hours. 20 worms were picked, treated with 200 mg/mL gentamycin for 3 hours to eliminate surface-bound bacteria and washed thoroughly using S-basal with 1 mM of sodium azide to remove any residual antibiotic. Washed nematodes were suspended in S-basal + 0.1% Triton-X and lysed by mechanical disruption using glass beads as described previously [19]. Appropriate dilutions of the lysates were plated onto LB agar with the appropriate antibiotics, to obtain bacterial counts. In order to visualize the bacterial accumulation within nematode guts, worms were fed *S. flexneri* strains tagged with GFP^+^. Following 24 hours of infection bacterial fluorescence was observed using the EVOS digital inverted microscope (AMG).

### Transmission electron microscopy

50–100 adult worms were picked off virulent and avirulent *S. flexneri* lawns 24, 96 and 144 hours post infection. For prolonged infection periods, worms were transferred onto fresh *S. flexneri* lawns each day. Worms were transferred into Beem capsules and fixed, rinsed and stained using the microwave-assisted irradiation protocol developed by Hall *et al.* [53]. A Pelco Biowave oven at the Centre of Advanced Microscopy (CAM, ANU), was used for the microwave assisted fixation of worms. Post staining with 0.5% aqueous uranyl acetate, samples were dehydrated at room temperature using the following dehydration cycles; 50% ethanol for 10 minutes, 70% ethanol for 10 minutes, 80% ethanol for 10 minutes, 90% ethanol for 10 minutes, three treatments with 100% ethanol for 10 minutes each. This was followed by infiltration of the sample at room temperature with LR White resin (Pro-SciTech) using the following infiltration regime: 2∶1 100% ethanol: LR White; 2 hours, 1∶1 100% ethanol: LR White; 2 hours, two treatments with 100% LR White for 2 hours each. LR white was cured at 65°C under nitrogen gas overnight. Thin sections were obtained using a Power Tome XL ultramicrotome (RMC, Boekeler Instruments, Tucson, AZ) at the Albert Einstein College of Medicine (AECOM). Sections were collected on copper slot grids and stained with 2% uranyl acetate in 50% ethanol for 10 minutes and with lead citrate (Reynolds’s formulation) for 15 minutes and TEM micrographs were collected on a Phillips CM10 electron microscope at AECOM.

### Isolation of total nematode protein

Approximately 500,000 synchronized, young adult nematodes were infected with *E. coli* OP50 (control) and wild type *S. flexneri* 3b (SFL1520) for 24 hours at 22°C. Post infection the nematodes were washed thoroughly using sterile S-basal with 1 mM sodium azide and treated with 200 mg/mL gentamycin for 3 hours to reduce the presence of bacterial spots on the gels. Sucrose flotation [49] was used to separate adult worms from larvae, eggs and bacterial debris. Infected worms were snap-frozen in liquid nitrogen and the frozen pellets were ground to a fine powder using fine glass powder in an ice-cold mortar and pestle and resuspended in solubilisation buffer (7 M urea, 2 M thiourea, 30 mM tris-base, 4% (w/v) CHAPS and Complete Protease Inhibitor (Roche)). The suspensions were homogenized and sonicated. Nematode proteins precipitated using 100% trichloroacetic acid (TCA) were collected by centrifugation, washed thrice using ice-cold acetone, air dried and resuspended in 20–30 µl 0.2 M NaOH and 100–200 µl of solubilization buffer (9 M urea, 4% CHAPS, 1% DTT, 1% ampholytes, 35 mM tris base). Protein concentration was determined using the Bradford method [54] and 2 mg of each sample was labeled with fluorescent dyes Cy3 or Cy5 (GE Healthcare). An internal standard, consisting of 1 mg of each sample, was labeled with Cy2.

### 2-D electrophoresis

2-D electrophoresis was performed in darkness to maintain the stability of the Cy dyes. Immobiline pH 3–10 NL Drystrips (24 cm, GE Healthcare) were used for the first dimension isoelectric focusing (IEF). The strips were rehydrated overnight in rehydration solution containing, 8 M urea, 0.5% (w/v) CHAPS, 0.2% (w/v) DTT, 0.52% (w/v) bio-ampholytes and 0.6% (w/v) bromophenol blue. Proteins from *S. flexneri* infected and control worms along with internal controls were loaded onto rehydrated Immobiline strips and IEF was carried out in a Multiphor II electrophoresis system (GE Healthcare) at 20°C for a total of 35,000 volt hours [55].

For separation of proteins across the second dimension, self-cast 12.5% SDS-PAGE gels were prepared using the EttanDALTsix system (GE Healthcare). The gels were cast using low fluorescence glass plates, which are compatible with visualizing the CyDyes. Focused first dimension strips were equilibrated as described by [55] and placed on the second dimension gels. SDS-PAGE was carried out at 10°C in SDS running buffer (25 mM Tris, pH 8.0, 192 mM glycine and 0.1% SDS) at 600 V, 10 mA, and 2.5 W per gel for the first hour; 600 V, 40 mA, and 13 W per gel until the bromophenol blue front reached the bottom of the gel.

### Gel imaging and image analysis

After the second dimension electrophoresis, DIGE-labeled proteins were visualized using a Typhoon Trio laser scanner (GE Healthcare). Gels were scanned with the specific excitation wavelengths of Cy3 (532-nm laser and a 580-nm band pass 30 emission filter), Cy5 (633-nm laser and a 670-nm band pass 30 emission filter) and Cy2 (488-nm laser and a 580-nm band pass 40 emission filter). Spot detection and analysis was carried out using DeCyder Version 6.5 (GE Healthcare) software followed by careful manual confirmation and rematching of matching errors. Statistics and identification of differentially expressed spots were carried out in the DeCyder DIA and BVA modules (one-way ANOVA).

### In-gel trypsin digestion of protein spots and liquid chromatography-mass spectrometry

After analysis of gel images, identified spots of interest were excised from the 2D-DIGE gels using an Ettan spot picker (GE Healthcare). In-gel trypsin digest was carried out as described by Mathesius *et al* [55] with a few modifications. Briefly, excised protein spots were washed four times in acetonitrile: 50 mM ammonium bicarbonate (49.5∶49.5, v/v). Spots were dried in 100% acetonitrile for 30 minutes following which they were air-dried to eliminate all acetonitrile. Gel pieces were rehydrated with a trypsin solution (20 units; Promega) and incubated for 2 hours at 4°C followed by overnight incubation at 37°C. Peptides were extracted from the gel pieces using an extraction buffer consisting of acetonitrile: water: trifluoroacetic acid (TFA) (50%:50%:1%, v/v) followed by gentle sonication in a sonic water bath for 40 minutes. Peptides were collected and dried completely to remove all traces of TFA and resuspended in 20 µl of acetonitrile: water: formic acid (10%:89.9%:0.1%, v/v). The peptides were identified at the Mass Spectrometry Facility, The Australian National University, on an Agilent 6530 Q-TOF LC/MS (Santa Clara, CA, USA) with a ChipCube ion source interface (Agilent Technologies, Inc., Palo Alto, CA) containing a liquid chromatographic chip (ProtID-Chip-150(II), separation: 150 mm×75 µm, enrichment: 4 mm 40 nL, packed with 5 µm Zorbax 300SB-C18 particles). The LC separation system included a binary capillary pump operated at a flow rate of 4 µL/min, used for loading the samples, and a nanoflow gradient pump using a linear gradient from 8 to 38% mobile phase B in 47 min at a flow rate of 300 nL/min. The column was then washed with 90% mobile phase B for 5 min. Mobile phase A was 0.1% formic acid and mobile phase B was 90% acetonitrile/water containing 0.1% formic acid.

The spray from the chip was subjected to positive polarity electrospray ionisation (ESI) using the following settings: gas flow rate 4 L/min, gas temperature 300°C, capillary voltage 1900 V, fragmentor 175 V, skimmer 65 V and octopole RF peak 750 V. The instrument was run in extended dynamic range mode with data dependent acquisition switching between MS (*m/z* 100–1700 at 3 spectra/s) and MS/MS (*m/z* 50–1700 at 3 spectra/s), measuring the collision induced dissociation (CID) fragment spectra of the three most intense precursor ions with charge states 2, 3 and ≥3 with a 15 s dynamic exclusion time, within a cycle time of 1.4 s. The collision energy was automatically set by the Agilent MassHunter Acquisition software (slope 3, offset 2). The *m*/*z* values of all ions present in the mass spectra were corrected against two reference ions (purine, [MH]^+^ *m*/*z* 112.985587 and 1H, 1H, 3H tetra(fluoropropoxy)phosphazine, [MH]^+^ *m/z* 922.0097). Data were acquired and analysed with Agilent Technologies MassHunter software (version B.4.0).

Proteins were identified through peptide sequences using MASCOT (Matrix Science). One missed cleavage per peptide was allowed and a mass tolerance between 0.3 and 0.1 Da was used for high stringency searches and 0.8 and 0.6 Da was used for low stringency searches. Carbamidomethylation of cysteine was set as a fixed modification and oxidation (M) as a variable. We searched the SwissProt database under taxonomy *C. elegans* on the MASCOT database at the Australian Proteomic Computation Facility (APCF).

### Quantitative real-time polymerase chain reaction (qRT-PCR)

Approximately 50,000 synchronized young adult worms were infected with either *E. coli* OP50 or *S. flexneri* serotype 3b for 24 h at 22°C. Adult worms were separated from eggs and bacterial debris using sucrose flotation [49]. Infected adult worm pellets were snap frozen in liquid nitrogen and RNA was isolated using Trizol reagent (Invitrogen) according to the manufacturer’s instructions. 200 ng of total RNA was used as template in a first strand synthesis using random hexamer primers and SuperScript II (Invitrogen) according to the manufacturer’s instructions. cDNA was used to set up qRT-PCRs using the power SYBR Green RT-PCR kit (Applied Biosystems) according to the manufacturer’s instructions except that primers were used at a final concentration of 0.4 µM and the final reaction volume was reduced to 10 µl. Results of qRT-PCRs represent 3 independent biological repeats. Reactions were run in a Rotor-Gene Q Real-Time cycler (Qaigen) and data were analysed using the Rota-Gene Q series software package.

## Supporting Information

Figure S1 **Two-dimensional differential in-gel electrophoresis of the** ***C. elegans*** **infected with** ***S. flexneri.***
(DOCX)

Figure S2 **Graphical representation of the predicted biological functions of the up- and down-regulated** ***C. elegans*** **proteins identified in response to** ***S. flexneri*** **infection.**
(DOCX)

Table S1 **Predicted** ***S. flexneri-*** **induced responses in** ***C. elegans*** **identified through Peptide Mass Fingerprinting using low stringency MASCOT search parameters.**
(DOCX)
