# Supplementary material for: Shigella flexneri Infection in Caenorhabditis elegans: Cytopathological Examination and Identification of Host Responses
Source: PLoS One. 2014 Sep 4;9(9):e106085. doi: 10.1371/journal.pone.0106085 (PMC4154869; doi:10.1371/journal.pone.0106085)
Supplement: Figure S1 — Two-dimensional differential in-gel electrophoresis of the C. elegans infected with S. flexneri. (DOCX) [file pone.0106085.s001.docx]

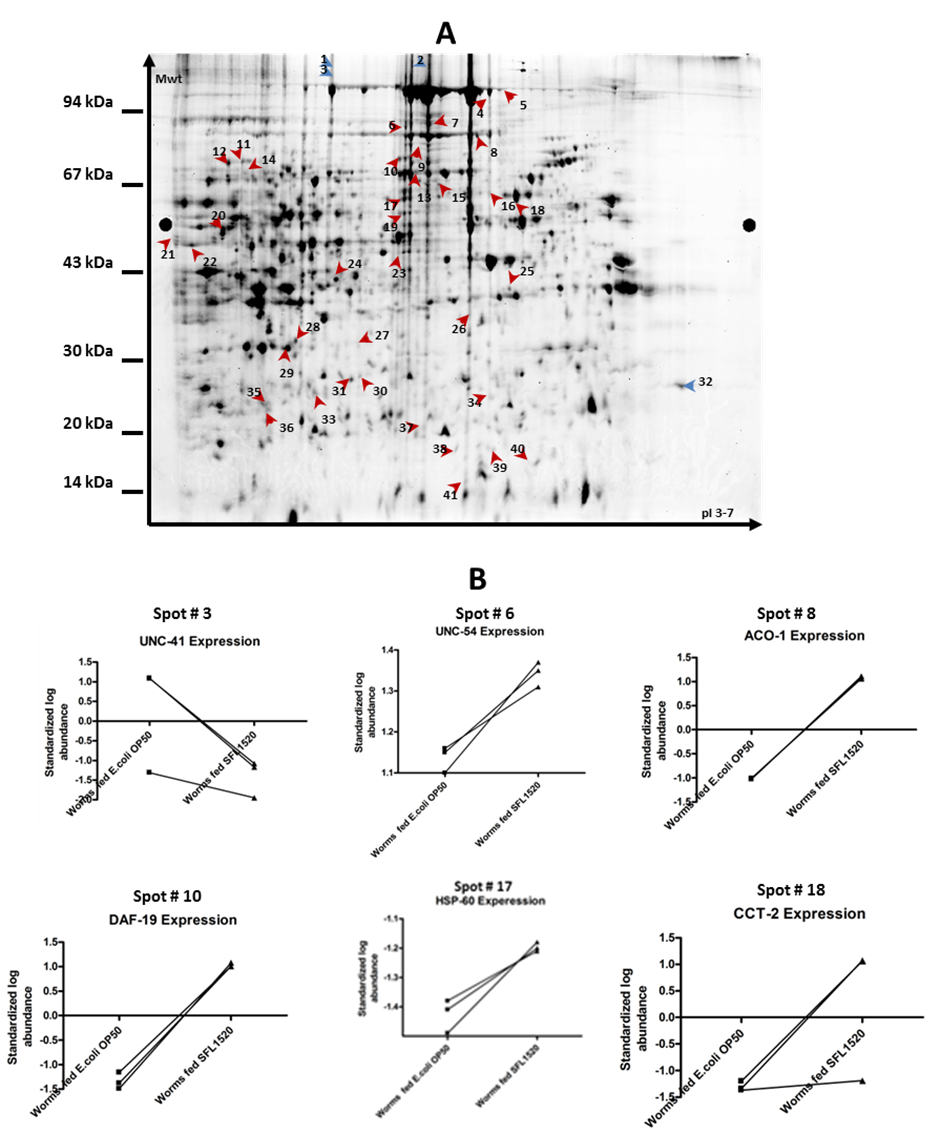


**Figure S1:** **Two-dimensional differential in-gel electrophoresis of the *C. elegans* infected with *S. flexneri.* A:** A representative 2 dimensional gel electrophoresis image of the *S. flexneri-*induced proteome of *C. elegans.* Red arrows indicate the protein spots identified to be up-regulated and blue arrows identify the spots identified as down-regulated in response to *S. flexneri* infection. All numbered spots were excised from the gel and submitted for liquid chromatography mass spectrophotometry analysis. Proteins were identified through peptide sequencing using MASCOT (Matrix Science) (see Tables 1 and S1). **B:** Differentially expressed spots identified using high stringency MASCOT search parameters. Graphs depicting the differences in standardized log abundance of identified spots in worms infected with virulent *S. flexneri* and control worms maintained on *E. coli* OP50 across three biological replicates calculated using the DeCyder version 5 software package.
