# Supplementary material for: Shigella flexneri Infection in Caenorhabditis elegans: Cytopathological Examination and Identification of Host Responses
Source: PLoS One. 2014 Sep 4;9(9):e106085. doi: 10.1371/journal.pone.0106085 (PMC4154869; doi:10.1371/journal.pone.0106085)
Supplement: Figure S2 — Graphical representation of the predicted biological functions of the up- and down-regulated C. elegans proteins identified in response to S. flexneri infection. (DOCX) [file pone.0106085.s002.docx]

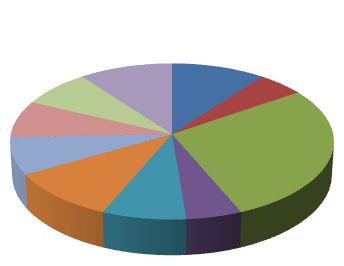


Enzymes

Replication

Transcription, translation, ribosomal structures

Transport

Chaperons

Locomotion and pharyngeal pumping

Cell division and reproduction

Innate immune response

Unknown

Others


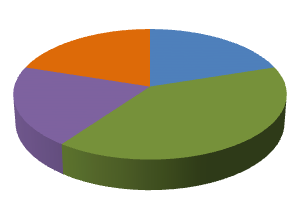


**Up regulated proteins**

**Down regulated proteins**

**Figure S2:** **Graphical representation of the predicted biological functions of the up- and down-regulated *C. elegans* proteins identified in response to *S. flexneri* infection.**
