# Supplementary material for: Shigella flexneri Infection in Caenorhabditis elegans: Cytopathological Examination and Identification of Host Responses
Source: PLoS One. 2014 Sep 4;9(9):e106085. doi: 10.1371/journal.pone.0106085 (PMC4154869; doi:10.1371/journal.pone.0106085)
Supplement: Table S1 — Predicted S. flexneri- induced responses in C. elegans identified through Peptide Mass Fingerprinting using low stringency MASCOT search parameters. (DOCX) [file pone.0106085.s003.docx]

**Table S1: Predicted *S. flexneri-*induced responses in *C. elegans* identified through MS/MS spectra using low stringency MASCOT search parameters.**

| **Spot No.** | **Pathogen-induced change in expression** | **Identified protein** | **UniProt Id** | **Gene Name** | **Biological function/relevant RNAi phenotypes** | **MASCOT Score** | **Theoretical Mwt (Da)/pI** | **Observed Mwt (Da)/pI** | **Sequence coverage** | **No of peptides matched** |
| --- | --- | --- | --- | --- | --- | --- | --- | --- | --- | --- |
| 1 | Down-regulated | Vacuolar protein sorting-associated protein 26 | VPS26_CAEEL | *vps-26* | Protein transport/ Premature death | 25 | 40926/5.81 | >94000/4.8 | 4% | 2 |
| 2 | Down-regulated | Uncharacterized NTE family protein | YOL_CAEEL | ZK370.4 | [Predicted to be involved in phosphatidylcholine metabolic process/ No](http://www.ebi.ac.uk/QuickGO/GTerm?id=GO:0046470) RNAi phenotypes specified | 27 | 153212/6.53 | >94000/5.2 | 2% | 4 |
| 3 | Down-regulated | Putative stoned B-like protein | STNB_CAEEL | *unc-41* | Locomotion and endocytosis/ defective movement, nervous system morphology variant | 27 | 190581/4.81 | >94000/4.8 | 3% | 7 |
| 4 | Up-regulated | UPF0533 protein | TPC13_CAEEL | C56C10.7 | Probable trafficking protein/ No RNAi phenotypes specified | 29 | 45329/5.48 | >94000/5.6 | 3% | 2 |
| 5 | Up-regulated | Probable cation-transporting ATPase | YE56_CAEEL | C10C6.6 | ATP catabolic process/ Sparse or enlarged yolk granules | 31 | 133598/7.25 | >94000/5.8 | 2% | 5 |
| 6 | Up-regulated | Myosin-4 | MYO4_CAEEL | *unc-54* | Locomotion, pharyngeal pumping/ bag of worms, egg laying defect, sluggish, premature death | 134 | 225958/5.59 | ~96000/5.2 | 8% | 14 |
| 7 | Up-regulated | Sterile alpha and TIR motif-containing protein | SARM1_CAEEL | *tir-1* | Innate immunity, neurogeneration, differentiation/ hypersensitivity to infection, reduced gene expression | 30 | 113781/6.9 | ~97000/5.3 | 1% | 3 |
|  |  | RING finger protein5 | RNF5_CAEEL | *rnf-5* | E3 ubiquitin ligase/ extended lifespan | 30 | 25298/6.22 | ~97000/5.3 | 9% | 1 |
| 8 | Up-regulated | Probable cytoplasmic aconitate hydratase | ACOC_CAEEL | *aco-1* | Cellular Iron homeostasis, TCA/ No RNAi phenotypes specified | 86 | 97113/5.49 | ~95000/5.5 | 14% | 8 |
|  |  | Elongation factor2 | EF2_CAEEL | *eef-2* | Protein biosynthesis/ apoptosis reduced, extended lifespan | 72 | 95477/6.1 | ~95000/5.5 | 18% | 9 |
| 9 | Up-regulated | DNA topoisomerase1 | TOP1_CAEEL | *top-1* | DNA replication/ shortened lifespan, defective locomotion | 29 | 94428/9.04 | ~93000/5.2 | 2% | 4 |
| 10 | Up-regulated | RFX-like transcription factor | DAF19_CAEEL | *daf-19* | Transcription factor/ cilia absent | 24 | 91421/5.97 | ~86000/5.1 | 2% | 3 |
| 11 | Up-regulated | Uncharacterized protein | YAO6_CAEEL | F54D1.6 | Unknown/ animals produce no or few embryos | 25 | 164864/6.15 | ~90000/4.5 | 3% | 7 |
|  |  | Nipped-B-like protein pqn-85 | NPBL_CAEEL | *pqn-85* | Cell cycle/ defective locomotion | 24 | 253743/5.79 | ~90000/4.5 | 4% | 8 |
| 12 | Up-regulated | DCT-5 | DCT5_CAEEL | *dct-5* | Acts downstream of Daf16/FOXO/ induced dauer formation | 26 | 24045/8.06 | ~90000/4.3 | 9% | 2 |
| 13 | Up-regulated | Uncoordinated protein79 | UNC79_CAEEL | *unc-79* | Locomotion/ sluggish, odour chemocensory response variant | 31 | 98899/6.07 | ~85000/5.2 | 6% | 5 |
| 14 | Up-regulated | Probable FAD synthase | FLAD1_CAEEL | R53.1 | FAD biosysnthesis/ slow growth | 28 | 59440/6.49 | ~85000/4.45 | 5% | 4 |
| 15 | Up-regulated | Uncharacterized protein | YKK6_CAEEL | C02F5.6 | HEN1 (RNA 3'end methyltransferase) of Nematode/ No RNAi phenotypes specified | 29 | 51993/5.23 | ~78000/4.4 | 1% | 1 |
| 16 | Up-regulated | Calcium-activated potassium channel | SLO1_CAEEL | *slo-1* | Potassium transport/ irregular pharyngeal pumping, desensitization to chemo attractants | 25 | 131043/5.8 | ~72000/5.6 | 2% | 4 |
| 17 | Up-regulated | Chaperonin homolog | CH60_CAEEL | *hsp-60* | Protein refolding, embryo larva development/ maternal sterile | 1446 | 60235/5.3 | ~68000/5.1 | 49% | 7 |
| 18 | Up-regulated | T-complex protein 1 subunit beta | TCPB_CAEEL | *cct-2* | Protein folding/ apoptosis reduced, maternal sterile, oocytes lack nuclei, resistant to oxidative stress-animals fail to respond to oxidative stress, endocytosis defective | 41 | 57338/5.65 | ~67500/5.6 | 6% | 2 |
| 19 | Up-regulated | E3 ubiquitin-protein ligase | RPM1_CAEEL | *rpm-1* | Ubiquitin protein ligase that negatively regulates the p38 MAPK pathway expressed in pharynx / apoptosis reduced | 28 | 425202/6.29 | ~67900/5.1 | 1% | 6 |
| 20 | Up-regulated | Muscle M-line assembly protein | UNC89_CAEEL | *unc-89* | Structural component of muscle M-line/ egg laying defective, locomotion defective, pharyngeal pumping reduced | 24 | 8990003/5.42 | ~60000/4.3 | 2% | 25 |
| 21 | Up-regulated | Mediator of RNA polymerase II transcription subunit1.1 | MED1_CAEEL | *sop-3* | Transcription regulation/slow growth, reduced brood size | 28 | 165015/8.86 | ~57000/4.0 | 2% | 3 |
| 22 | Up-regulated | Heat shock 70 kDa protein F | HSP7F_CAEEL | *hsp-6* | Stress response/ reduced ATP, reduced pharyngeal pumping, shortened lifespan, reduced brood size | 24 | 71086/5.89 | ~55000/4.1 | 3% | 3 |
| 23 | Up-regulated | Twitchin | UNC22_CAEEL | *unc-22* | Regulates muscle contraction and relaxation, expressed in the body wall, vulva, pharynx/ defective movement | 49 | 792459/5.79 | ~50000/5.1 | 1% | 12 |
| 24 | Up-regulated | Ribosome biogenesis regulatory protein homolog | RRS1_CAEEL | *rrbs-1* | Ribosome biogenesis/maternal sterile, reduced oocytes, | 24 | 37962/9.97 | ~44000/4.9 | 6% | 3 |
| 25 | Up-regulated | Uncharacterized protein | YMF7_CAEEL | F55H2.7 | Unknown | 29 | 44124/10.08 | ~42000/5.4 | 6% | 3 |
|  |  | Nuclear hormone receptor family member-77 | NHR77_CAEEL | *nhr-77* | Transcription regulation/ No RNAi phenotypes specified | 25 | 42688/9.12 | ~42000/5.4 | 11% | 3 |
| 26 | Up-regulated | Copine family protein 2 | CPNA2_CAEEL | *cpna-2* | Belongs to the copine family and is expressed in the body wall muscles/ No RNAi phenotypes specified | 25 | 862013/5.21 | ~40000/5.5 | 1% | 17 |
|  |  | Regulator of nonsense transcripts1 | RENT1_CAEEL | *smg-2* | RNA dependent helicase, RNA interference, embryo genital morphogenesis/ defective mRNA surveillance, reduced susceptibility to RNAi | 25 | 121142/6.46 | ~40000/5.5 | 2% | 3 |
|  |  | Germline survival defective-1 | GLS1_CAEEL | *gls-1* | Meiosis/ defective oogenesis | 18 | 116684/8.44 | ~40000/5.5 | 7% | 5 |
| 27 | Up-regulated | N-terminal acetyltransferase B complex subunit NAA25 homolog | NAA25_CAEEL | *cra-1* | Catalyses acetylation of proteins/reduced number of oocytes and brood size | 27 | 110296/6.41 | ~32000/4.9 | 2% | 4 |
| 28 | Up-regulated | No significant hits |  |  |  |  |  |  |  |  |
| 29 | Up-regulated | No significant hits |  |  |  |  |  |  |  |  |
| 30 | Up-regulated | Protein arginine N-methyltransferase 5 | ANM5_CAEEL | *prmt-5* | Transcription regulation/ Increased spontaneous mutation | 34 | 83582/5.86 | ~29000/4.9 | 2% | 3 |
| 31 | Up-regulated | Nuclear hormone receptor family member-19 | NHR19_CAEEL | *nhr-19* | Transcription regulation/ No RNAi phenotypes specified | 33 | 541000/9.06 | ~28500/4.87 | 5% | 3 |
| 32 | Down-regulated | Single-stranded DNA-binding protein, mitochondrial | MTSS1_CAEEL | *mtss-1* | DNA replication/ reduced hypoxia response | 27 | 19227/9.77 | ~28000/6.7 | 8% | 1 |
|  |  | Histone H3-like centromeric protein | CPAR1_CAEEL | *cpar-1* | Nucleosome assembly/ reduced brood size | 24 | 29125/7.94 | ~28000/6.7 | 6% | 2 |
| 33 | Up-regulated | Twitchin | UNC22_CAEEL | *unc-22* | Regulates muscle contraction and relaxation, expressed in the body wall, vulva, pharynx/ defective movement | 29 | 792459/5.79 | ~25000/4.7 | 1% | 19 |
|  | Up-regulated | Homeobox protein | HM31_CAEEL | *ceh-31* | Multicellular organism development/ intestinal cell proliferation variant | 28 | 28834/7.82 | ~25000/4.7 | 5% | 2 |
| 34 | Up-regulated | Origin recognition complex subunit 2 | ORC2_CAEEL | *orc-2* | DNA replication/ sluggish | 25 | 49460/6.03 | ~26000/5.6 | 5% | 3 |
| 35 | Up-regulated | UPF0046 protein | YW12_CAEEL | T07D4.2 | Hydrolase activity/ No RNAi phenotypes specified | 26 | 45353/9.17 | ~24000/4.6 | 9% | 3 |
| 36 | Up-regulated | Nuclear hormone receptor family member-77 | NHR77_CAEEL | *nhr-77* | Transcription regulation/ No RNAi phenotypes specified | 29 | 42688/9.12 | ~23000/4.6 | 4% | 3 |
| 37 | Up-regulated | 26S proteasome non-ATPase regulatory subunit 6 | PSMD6_CAEEL | *rpn-7* | ATP-dependent degradation of ubiquitinated proteins/ Increased apoptosis | 31 | 47838/6.36 | ~21000/5.2 | 1% | 1 |
| 38 | Up-regulated | Mediator of RNA polymerase II transcription subunit 17 | MED17_CAEEL | *mtd-17* | Transcription regulation/ defective egg laying, locomotion, endocytosis | 27 | 77767/5.74 | ~20000/5.5 | 2% | 3 |
| 39 | Up-regulated | Probable cation-transporting ATPase | YE56_CAEEL | C10C6.6 | ATP catabolic process/ Sparse or enlarged yolk granules | 33 | 133598/7.25 | ~20000/5.6 | 2% | 4 |
| 40 | Up-regulated | 26S proteasome non-ATPase regulatory subunit 3 | PSMD3_CAEEL | *rpn-3* | Regulation of protein catabolic process/ increased apoptosis | 24 | 57813/7.14 | ~18000/5.8 | 5% | 3 |
| 41 | Up-regulated | Cytoplasmic polyadenylation element-binding protein 1 | CPB1_CAEEL | *cpb-1* | Differentiation, spermatogenesis/ Reduced brood size | 30 | 63949/8.65 | ~13000/5.5 | 1% | 1 |
